# A Symbols Based BCI Paradigm for Intelligent Home Control Using P300 Event-Related Potentials

**DOI:** 10.3390/s222410000

**Published:** 2022-12-19

**Authors:** Faraz Akram, Ahmed Alwakeel, Mohammed Alwakeel, Mohammad Hijji, Usman Masud

**Affiliations:** 1 Department of Biomedical Engineering, Riphah International University, Islamabad 46000, Pakistan; 2 Sensor Networks and Cellular Systems Research Center, University of Tabuk, Tabuk 71491, Saudi Arabia; 3 Faculty of Computers & Information Technology, University of Tabuk, Tabuk 71491, Saudi Arabia; 4 Faculty of Electrical and Electronics Engineering, University of Engineering and Technology, Taxila 47050, Pakistan; 5 Department of Electrical Communication Engineering, University of Kassel, 34127 Kassel, Germany

**Keywords:** brain-computer interface, smart home, phone control, event-related potentials, EEG, P300

## Abstract

Brain-Computer Interface (BCI) is a technique that allows the disabled to interact with a computer directly from their brain. P300 Event-Related Potentials (ERP) of the brain have widely been used in several applications of the BCIs such as character spelling, word typing, wheelchair control for the disabled, neurorehabilitation, and smart home control. Most of the work done for smart home control relies on an image flashing paradigm where six images are flashed randomly, and the users can select one of the images to control an object of interest. The shortcoming of such a scheme is that the users have only six commands available in a smart home to control. This article presents a symbol-based P300-BCI paradigm for controlling home appliances. The proposed paradigm comprises of a 12-symbols, from which users can choose one to represent their desired command in a smart home. The proposed paradigm allows users to control multiple home appliances from signals generated by the brain. The proposed paradigm also allows the users to make phone calls in a smart home environment. We put our smart home control system to the test with ten healthy volunteers, and the findings show that the proposed system can effectively operate home appliances through BCI. Using the random forest classifier, our participants had an average accuracy of 92.25 percent in controlling the home devices. As compared to the previous studies on the smart home control BCIs, the proposed paradigm gives the users more degree of freedom, and the users are not only able to control several home appliances but also have an option to dial a phone number and make a call inside the smart home. The proposed symbols-based smart home paradigm, along with the option of making a phone call, can effectively be used for controlling home through signals of the brain, as demonstrated by the results.

## 1. Introduction

A neurological disease known as a locked-in syndrome is caused by the total paralysis of all voluntary muscles throughout the body. It could be caused by brain or spinal cord damage, amyotrophic lateral sclerosis, brainstem stroke, diseases of the circulatory system, damage to nerve cells, and several other neuromuscular diseases. People who have locked-in syndrome are completely cognizant and able to reason and think, but they are unable to talk or move anything other than their eyes. All other voluntary muscle movement is blocked, making it impossible for them to speak or move [1,2,3]. Locked-in syndrome patients may generally move their eyes and can occasionally blink to communicate, but some seriously ill patients can also lose control of their eye movements and may become completely paralyzed. It is extremely difficult to interact with individuals who have locked-in syndrome and other forms of paralysis since they are unable to speak or express their needs or sentiments to those around them due to their lack of muscle-based modes of communication. For such patients, a direct brain-computer interface (BCI), which sends messages and instructions to the outside world via a new, non-muscular communication and control channel, may be an effective option. These people may be able to communicate once again because of brain-computer interfaces that open a new line of communication between their brain signals and computers. Recent research has demonstrated that BCI technology makes it viable for the brain to directly interact with the outside world, enabling paralyzed people to interact and gain control. The BCI technology enables users to interact with computers and operate appliances without using their muscles. BCI research aims to improve the overall quality of life for the disabled by giving them access to technology that allows them to interact with their environment. To attain this objective, several BCI applications have been presented in the literature. including character spelling [4,5], word typing [6], controlling a wheelchair [7,8], controlling a robotic/prosthetic limb [9], virtual reality control [10,11], neurorehabilitation [12], controlling a car [13], web browser control [14], Unmanned Aerial Vehicle (UAV) control [15,16], and games [17,18,19]. All these BCIs take commands from the brain and transform them to control signals for the desired application.

There are numerous ways of reading the brain’s activity such as functional Magnetic Resonance Imaging (fMRI), Magnetoencephalography (MEG), Positron Emission Tomography (PET), Computer Tomography (CT), Electrocorticography (ECoG), and functional Near-Infrared Spectroscopy (fNIRS). However, Electroencephalography (EEG) has been the most popular type for BCIs because it is non-invasive, relatively cheap, and easier to use. The brain’s EEG signal is made up of numerous signals with varied characteristics, each of which corresponds to a different mental activity. P300 Event-Related Potential (ERP), one of the signals, has been used in various BCI applications. When the user is exposed to an uncommon stimulus in a sequence of common stimuli, about 300 milliseconds after the start of the target/rare stimulus, a positive wave called P300 appears. It is induced when a person recognizes an occasional “target” stimulus among a regular stream of common “non-target” stimuli. When the user concentrates on a specific stimulus while simultaneously being exposed to several stimuli, P300 can be induced. In 1998, Farwell and Donchin [20] were the first to demonstrate a character spelling application of the P300-ERP. They proposed displaying a flashing matrix, having 6 rows and 6 columns of characters and numbers, to the users, and taking EEG signals of the users. They could get the P300 wave only after the target character was flashed, which could be detected using a classifier. The P300 signal has been used for numerous other applications of BCI; for instance, directing the movement of a cursor or a screen item [21,22], browsing the internet [23], file explorer [24], Playing games [25], and navigating a wheelchair [26].

There have been few studies on using the BCI to operate a smart-home environment. Hoffmann et al. [27] proposed an interface consisting of 6 images of home appliances for disabled subjects, and the users could select one of the images. Their proposed image flashing paradigm had been the most popular paradigm for smart-home control and several researchers used the same scheme. Achanccaray et al. [28] and Cortez et al. [29] used the same paradigm to control six home appliances. The problem with this image flashing paradigm is that the users have only six commands available, therefore they can perform only six tasks in a smart home. Carabalona et al. [30] developed an icon-based smart home control system. They proposed to use icons instead of characters in a smart home control system. However, the accuracy achieved using their proposed system was very low i.e., 50%. Park et al. [31] used Steady-State Visual Evoked Potentials (SSVEP) to operate three household appliances: a robotic vacuum cleaner, an air cleaner, and a humidifier. Kais et al. [32] proposed an implementation of home devices control using the motor imagery BCI. They used the BCI competition dataset where subjects performed four types of motor imagery (Left hand, Right hand, Foot, and Tongue). These four motor imagery states can be used to turn on/off two devices. Edlinger et al. [33] combined P300 and SSVEP for controlling a virtual home environment and tested their system with three subjects. Katyal et al. [34] also proposed a hybrid paradigm containing SSVEP and P300 to increase the number of decision options. Similarly, Chai et al. [35] proposed combining SSVEP with electromyography (EMG) signals. Uyanik et al. [36] proposed an SSVEP-based BCI to control a wheelchair along with a smart home. Kim et al. [37] combined ERPs with speech imagery. Usman et al. [38], proposed a symbols-based smart home control system using the P300. The suggested method used a 6×4 matrix, akin to the character spelling paradigm. The proposed system was tested on three subjects and achieved an accuracy of 87.5%. Taejun Lee et al. [39] utilized a BCI based on the P300 to control three home applications: the door lock, the electric light, and the speaker. Vega et al. [40] used six symbols of commonly used home appliances and employed a deep learning model for the classification of EEG. Shukla et al. [41,42] used a 2 × 3 matrix containing six symbols (TV, Mobile, Door, Fan, Light bulb, and Heater) and used P300-ERP to control these six appliances. They tested their system with 14 participants and achieved an average accuracy of 92.44%. The problem with such systems is the limited number of control options available to the users.

This paper proposes a P300-based intelligent home control system that allows users to control multiple home appliances and allows them to dial a phone call. Our interface comprises a matrix of twelve symbols. Each symbol indicates a smart home activity that can be executed. Users can choose any symbol to perform their desired action, such as turning on or off lights. To detect P300, we employed a supervised machine learning algorithm, Random Forest (RF), and evaluated our proposed system with 10 healthy participants for smart home control and making phone calls. The accuracy rate was 92.25 percent on average.

The rest of the paper is organized as follows. Section 2 presents a detailed methodology of the proposed home control system. The experimental results are presented in Section 3. Finally, Section 4 presents a conclusion and discussion.

## 2. Materials and Methods

**Figure 1:**
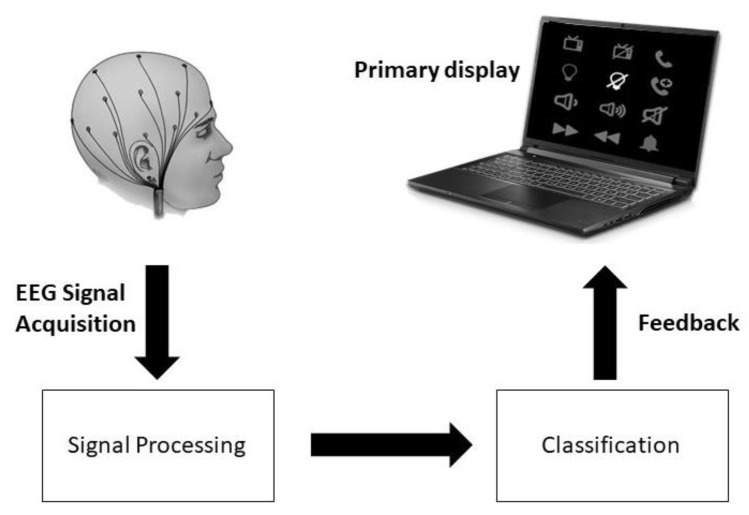
Block diagram of the proposed system.

Our methodology comprises displaying a flashing paradigm to the user as shown in Figure 1. While the user is observing the flashing paradigm, EEG waves are recorded using a 32-channel EEG cap. After preprocessing, a random forest classifier is then used to classify the P300 signal of the brain. The classification of the P300 signal leads to choosing the target symbol. Ideally, the P300 should be found after 300 ms of the flashing of the symbol the user is focusing on.

### 2.1. Participants

Ten healthy volunteers (7 males and 3 females; ages 18 to 35 years) participated in the experiments. The participants’ vision was normal or corrected and had no record of any neurological brain illnesses. None of the participants had any prior knowledge or experience with BCIs of any kind. The experimental protocol was explained to the participants and all participants signed a written informed consent form before the start of the experiment.

### 2.2. Primary Display for Controlling Home Appliances

**Figure 2:**
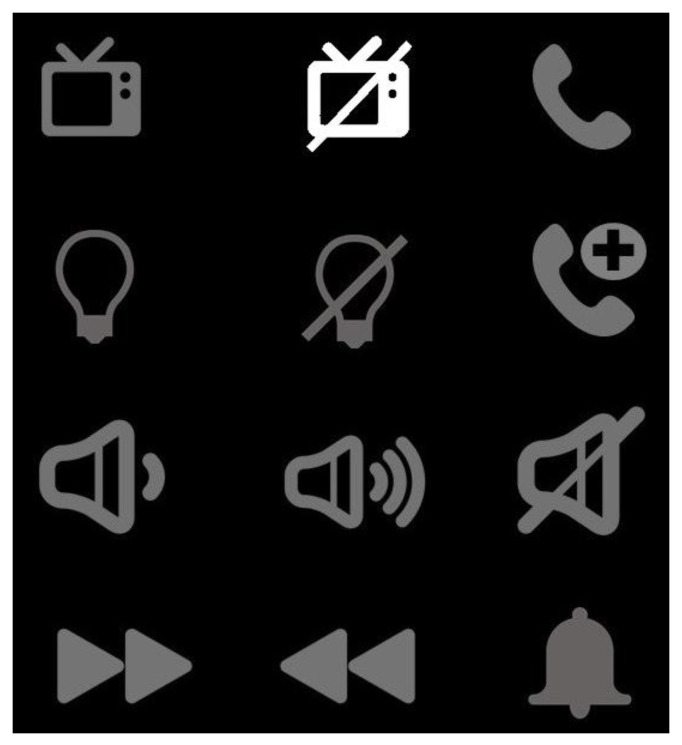
Primary Display to control the smart home.

**Figure 3:**
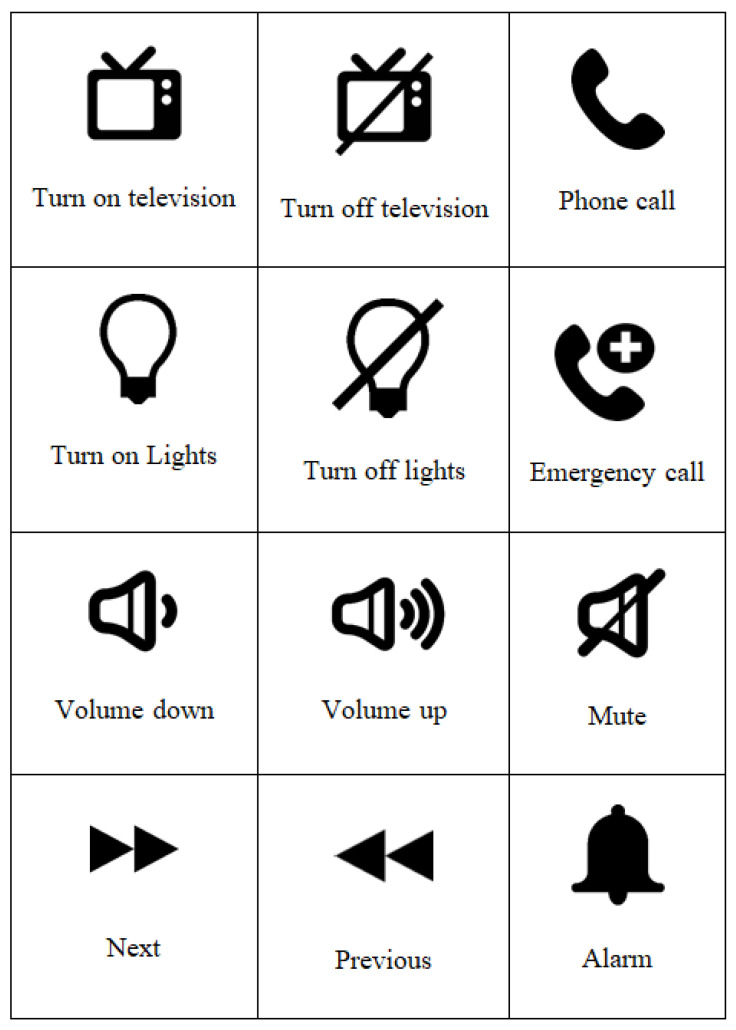
Functional symbols on the main Interface and their description.

In this paper, we propose an interface that consists of a 4 × 3 matrix containing 12 symbols. Each symbol is randomly intensified as shown in Figure 2, and the users must concentrate on the symbol they want to choose. The description of those symbols is shown in Figure 3. Rather than intensifying rows and columns of the matrix as in traditional P300 displays, we chose to intensify each symbol individually because previous research has shown that the prior probability of the target is inversely related to the amplitude of the P300 [43]. Higher probabilities of the appearance of the target lead to lower amplitudes of the P300-ERP, which in turn makes the classification difficult and hence reduces accuracy. In the case of our proposed smart home control, we have only 12 symbols in the main display making it a 4 × 3 matrix. Using the row and column-wise intensification will lead us to have a target probability of 2/7 because out of seven intensifications (4 rows and 3 columns) two should contain the target symbol (one row and one column). Whereas intensifying each symbol gives us the probability of the appearance of the target symbol to be 1/12 which is far less than the row/column intensification.

The subjects were told to randomly choose a symbol and focus on the chosen symbol and count how many times it flashed quietly. After 300 ms of the target symbol flashing, P300 appears. Intensifications are randomized in blocks. Each symbol is intensified exactly once in random order in each block of twelve intensifications. This intensification block is repeated fifteen times in total. We employed a 100 ms intensification time with a 75 ms blank interval in between.

On the primary display, the users have twelve symbols to choose from, and each symbol represents an action to be taken in the smart home such as controlling the TV, lights, volume, and phone. The description of all those symbols is shown in Figure 3.

### 2.3. Secondary Display for Making Phone Calls

**Figure 4:**
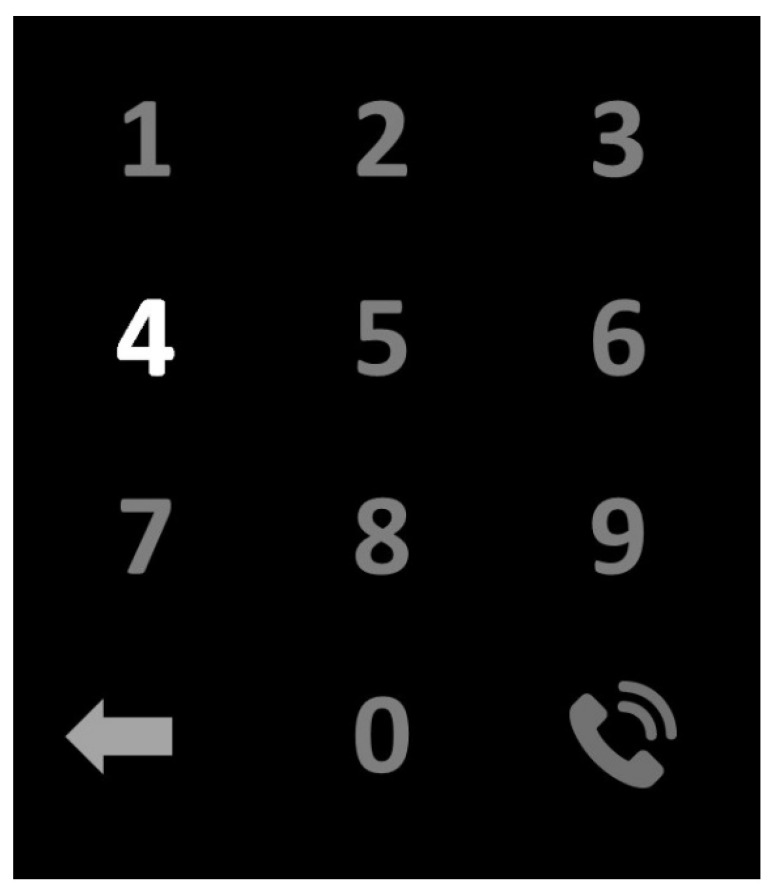
Secondary display for making phone calls.

In our proposed system, the users have the option of making phone calls along with controlling the home appliances. On the main display, there is a symbol for a phone call as shown in Figure 2 and Figure 3. If the user selects that phone call symbol, the display gets changed to a secondary display containing a 4 × 3 matrix of numbers that are shown in Figure 4. The secondary display contains numbers (0–9) for typing the phone number to be called, a call symbol for connecting the phone call, and a return symbol to disconnect the phone call and return to the primary display.

### 2.4. Experimental Setup

**Figure 5:**
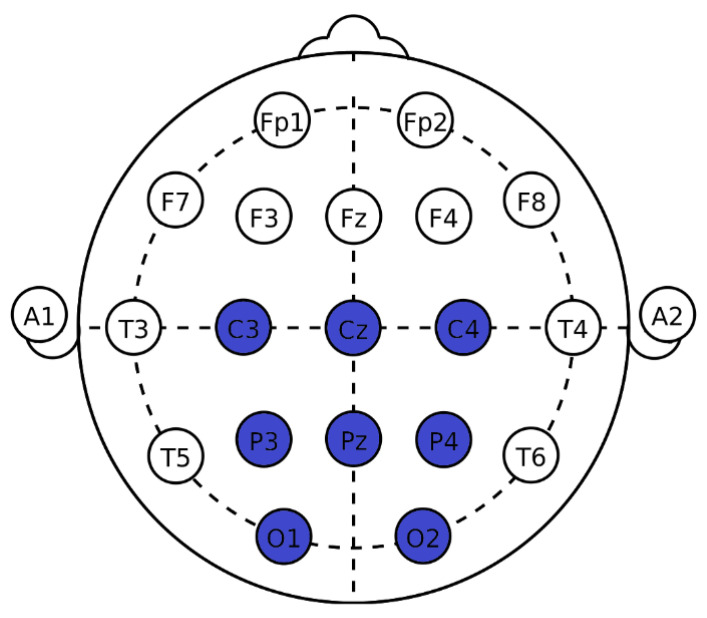
Electrode placement (highlighted electrodes are used in this study).

A 32-channel EEG data was acquired, as per the 10–20 international electrode system. The placement of electrodes is shown in Figure 5. Out of those 32 channels, only 8 were used for the classification of ERPs. The subjects were seated on a cozy chair in front of a display screen and were instructed to focus on the desired symbol from the displayed symbols (as shown in Figure 2). Each participant was required to attend two sessions: training and testing. Each participant was instructed to select a random symbol/number during the training session, and this experiment was repeated 10 times. During the test session, each participant chose 40 symbols/numbers at random (20 symbols on the main display and 20 numbers on the secondary display).

### 2.5. Signal Processing and Classification

In this study, we used 8 channels for signal processing and classification. The locations of the used electrodes are shown in Figure 5. It is known from the previous studies [44,45] that the P300-ERP is most dominant in the Pz area and the nearby electrodes. Therefore, we have chosen only those locations. The acquired EEG signals are bandpass filtered using a third-order Butterworth filter between 0.1 Hz to 25 Hz to remove the noise. The filtered signal is then segmented, and segments of 800 ms are extracted after the onset of each stimulus, i.e., flashing of each symbol from each of the used channels. To construct a single feature vector, the data segments are concatenated over the eight channels.

For the classification of P300-ERPs, we employed the random forest classifier because RF had shown superior performance in P300 classification in our previous studies [38,46]. We also compared the results of the random forest classifier with commonly used classification methods such as Support Vector Machine (SVM), Linear Discriminant Analysis (LDA), and k-nearest neighbors (kNN).

Random Forest is an ensemble classification technique. The general idea of the random forest is to build multiple decision tree classifiers and combine their results for better accuracy. Using multiple classifiers in an ensemble gives a more stable prediction. Random forest utilizes a random subset of the data to train each of the decision trees in the forest and then combines the result of each decision tree by majority voting. In this way, the weak classifiers are combined to form a strong classifier.

Random Forest was used to detect the presence of P300 ERPs in the recorded EEG signal. The system selects the symbol that elicits P300-ERP and displays the result as feedback to the user.

## 3. Results

We conducted experiments using our proposed paradigm on ten healthy volunteers. The 32 channels’ EEG data was acquired at a sampling frequency of 250 Hz. The participants were instructed to pay close attention to the target symbol and silently count how many times it flashed. This silent counting helps users in maintaining their attention. Each participant attended two sessions, namely training, and testing. During training, each participant selected 10 randomly selected symbols and during the test session, 40 symbols/numbers were selected, one at a time, as per the choice of the participants. The users were shown the main display, as shown in Figure 2. The symbols on the display were randomly flashing. The flashing time is shown in Table 1.

**Figure 6:**
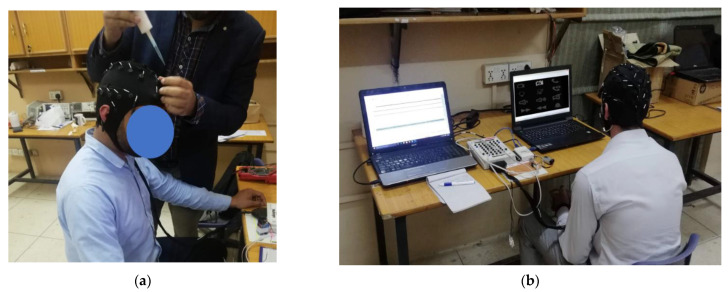
Data Collection. (**a**) Preparation for EEG data collection; (**b**) A participant during data collection.

Flashing a single symbol takes 175 ms: 100 ms for flashing and 75 ms of blank time in between two flashes. The proposed paradigm has a total of 12 symbols on the screen. Therefore, to flash each symbol once, the system requires 2.1 s. We had chosen to flash each symbol 15 times. Therefore, if we calculate the time required to select a symbol by each user, it comes out to be 31.5 s (2.1 × 15). The same timings were used in the second (numbers-based) paradigm to make a phone call. The users can select each number in 31.5 s. Figure 6 shows the images of the data collection.

### 3.1. Experimental Results

During the test session, each user was asked to select twenty random symbols on the primary symbols-based display, and twenty random numbers on the phone call interface. Table 2 presents the accuracy of classification for each subject on both displays. The random forest classifier achieved an overall accuracy of 92.25 percent.

We also compared the results of the random forest classifier with other commonly used classifiers such as SVM, LDA, kNN. The comparison of the accuracies obtained by these classifiers is presented in Table 3. All these classifiers performed well with minor differences in accuracies. RF classifier performed the best with an average accuracy of 92.25, whereas the SVM, LDA, and kNN achieved average classification accuracies of 91, 89.25, and 90.25 percent, respectively.

### 3.2. Waveform Morphologies

**Figure 7:**
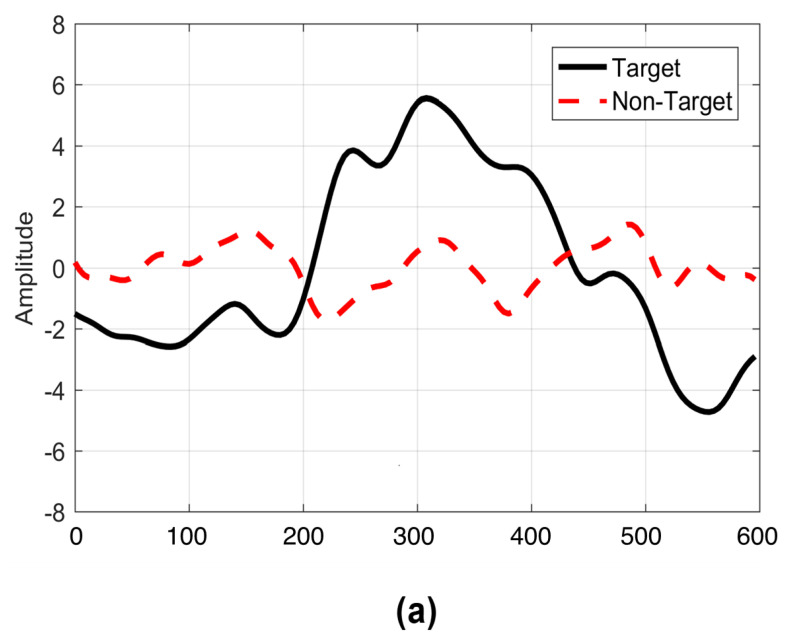

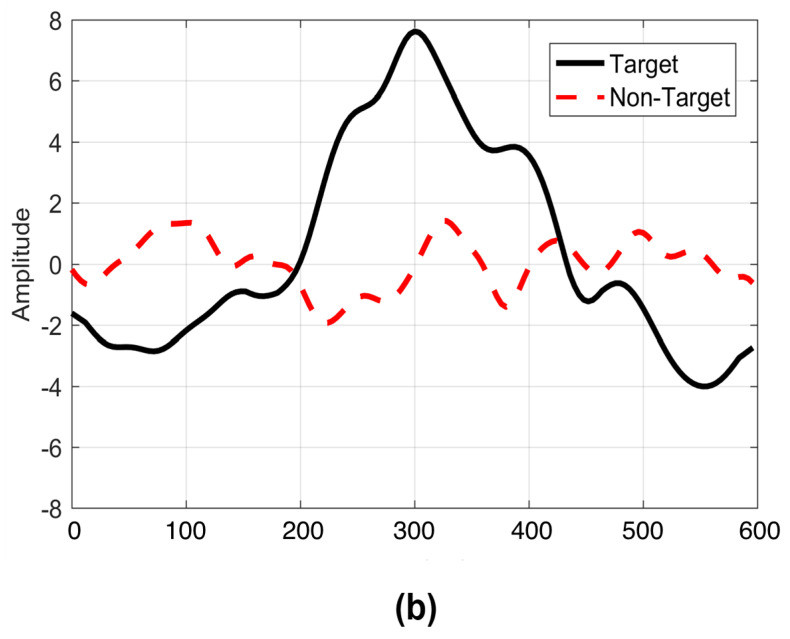
Waveforms for target and non−target stimuli. (**a**) Primary symbols−based display for controlling home appliances. (**b**) Secondary display (Numbers−based) for making phone calls.

The averaged ERPs for target and non-target stimuli are shown in Figure 7 for both the proposed displays. The P300 event-related potential is visible for the target cases in both paradigms. However, it was noted that the amplitude of the P300 was smaller in the case of the symbols-based paradigm. The accuracy was also lesser in the case of the symbols-based paradigm as shown in Table 2.

**Figure 8:**
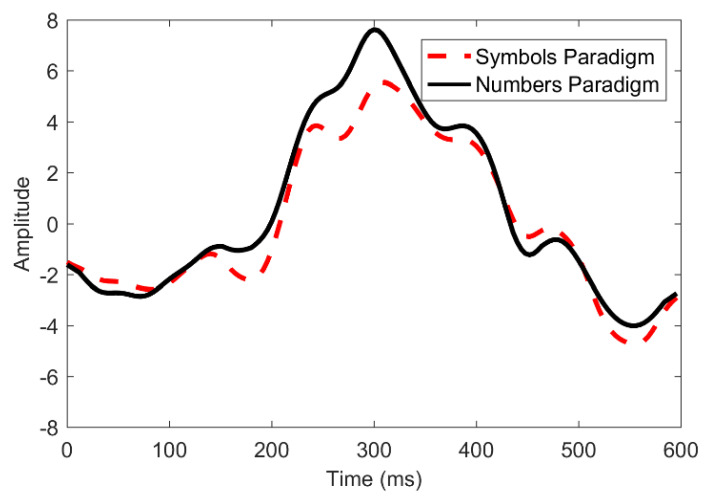
Comparison of the ERPs for both the displays.

**Figure 9:**
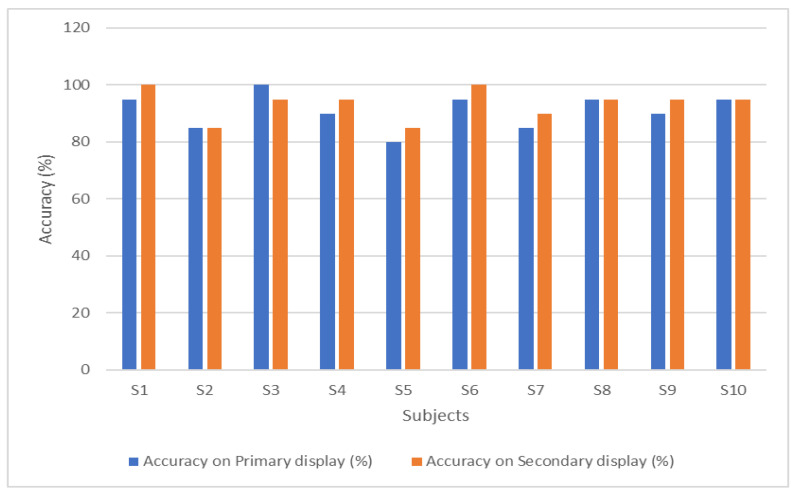
Comparison of the classification accuracies for both the displays.

A comparison of the P300 waveforms for the primary and secondary paradigms is shown in Figure 8. The amplitude of the numbers-based paradigm is larger than the symbols-based paradigm. The reason for this difference in P300 amplitude can be the familiarity of the users with numbers. The users are more familiar with the numbers, as compared to the symbols. This familiarity improves the user’s P300 response, which leads to improved classification accuracy. The comparison of accuracies on both the paradigms is presented in Figure 9.

A slight difference in accuracies of the primary and secondary display can be seen in Figure 9, because the amplitude of P300-ERPs was smaller in the case of the symbols-based paradigm. The numbers-based paradigm produced better P300 however, the difference is not that significant. On average, the numbers-based paradigm has 2.5% better accuracy than the symbols-based paradigm.

## 4. Discussion and Conclusions

This paper presented a novel BCI paradigm to control home appliances using the P300-ERP of the brain. The proposed paradigm included common household appliances such as televisions, lights, and music. We also included an option to make a phone call in our smart home. As evident from the results, the proposed P300-BCI system is capable of both controlling appliances and making phone calls in a smart home setting.

Compared to our previously proposed smart home system [38], we have achieved better accuracy in this paper. The reason for this improvement in accuracy is a single symbol flashing instead of flashing rows and columns. When we do rows/columns flashing in a 4 × 3 matrix, there are a total of 7 flashes (4 rows and 3 columns). Out of these seven flashes, two flashes correspond to the target symbol/number (one row and one column). This leads the prior probability of the target to be 2/7 or 0.286. Whereas flashing individual numbers/symbols in the same 4 × 3 matrix gives us a prior probability of target as 1/12 or 0.083 (one symbol out of a total twelve). It is known from previous studies [43] that in an oddball paradigm, the P300’s magnitude is negatively correlated with the target’s prior probability. The probability of flashing the target symbol is very low in this proposed paradigm as compared to the previous studies. Therefore, it leads to better P300 s, which in turn gives us better classification accuracy. Moreover, we have included a secondary paradigm for making phone calls and the users were able to dial phone numbers with an accuracy of 93.5%.

We can see from the results that the accuracy of the secondary display (numbers-based) is slightly better than the symbols-based display, one reason for this can be the familiarity of the users with the numbers as compared to the symbols. These results are consistent with Kaufmann et al. [47] where they had replaced the characters with the popular faces in a P300-based paradigm and achieved better accuracies.

Compared with the other smart home applications proposed in the literature, the proposed paradigm offers a more degree of freedom with an increased number of devices to control. The users can control 12 devices at a time using a single interface. The users also have the freedom to dial a phone number of their choice and make a phone call, which is an important aspect in improving the quality of life of the disabled. The number of symbols can further be increased and can be changed depending on the application and requirements of the users.

We have calculated the time required by the users to perform each command in the smart home. The users require 31.5 s to perform each command (selecting a symbol or a number). The time can be reduced by reducing the number of repetitions in flashing. However, if we reduce the number of flashes, the classification of P300 s gets difficult and may decrease the classification accuracy. In the future, better classification models can be employed to solve this problem of P300 recognition in a lesser number of repetitions.

## Figures and Tables

**Table 1:** Flashing time of the proposed paradigm.

Intensification time	100 ms
Inter-stimulus blank time	75 ms
Total Symbols	12
Number of repetitions for each symbol	15

**Table 2:** Classification accuracies on the proposed displays using random forest classifier.

Subjects	Accuracy on Primary Display (%)	Accuracy on Secondary Display (%)	Average Accuracy (%)
S1	95	100	97.5
S2	85	85	85
S3	100	95	97.5
S4	90	95	92.5
S5	80	85	82.5
S6	95	100	97.5
S7	85	90	87.5
S8	95	95	95
S9	90	95	92.5
S10	95	95	95
**Mean**	**91**	**93.5**	**92.25**

**Table 3:** Comparison of classification accuracies using RF, SVM, LDA, and kNN classifiers.

Subjects	Random Forest	SVM	LDA	kNN
S1	97.5	95	92.5	95
S2	85	85	82.5	85
S3	97.5	97.5	95	92.5
S4	92.5	95	90	92.5
S5	82.5	85	82.5	80
S6	97.5	92.5	90	92.5
S7	87.5	85	87.5	87.5
S8	95	95	92.5	90
S9	92.5	92.5	90	95
S10	95	87.5	90	92.5
**Mean**	**92.25**	**91**	**89.25**	**90.25**

## Abbreviations

The following abbreviations are used in this manuscript:

BCI	Brain-Computer Interface
CT	Computer Tomography
ECoG	Electrocorticography
EEG	Electroencephalography
EMG	Electromyography
ERP	Event Related Potential
fMRI	functional Magnetic Resonance Imaging
fNIRS	functional near-infrared spectroscopy
kNN	k-Nearest Neighbors
LDA	Linear Discriminant Analysis
MEG	Magnetoencephalography
PET	Positron Emission Tomography
RF	Random Forest
SSVEP	Steady-State Visual Evoked Potentials
SVM	Support Vector Machine
